# Strongyloidiasis in a Rural Setting — Southeastern Kentucky, 2013

**Published:** 2013-10-25

**Authors:** Stephanie Davis, Elizabeth Bosserman, Susan Montgomery, Dana Woodhall, Elizabeth S. Russell

**Affiliations:** Div of Parasitic Diseases and Malaria, Center for Global Health; EIS Officer, CDC

Strongyloidiasis is caused by *Strongyloides stercoralis,* a parasitic nematode (worm). Initial symptoms can include abdominal pain, diarrhea, or rash. Infection is often asymptomatic in the chronic phase but can be life-threatening in immunosuppressed persons. Transmission typically occurs when larvae from stool-contaminated soil penetrate skin; intraintestinal autoinfection is also possible, sometimes allowing infection to persist for decades. Serologic studies are often used in prevalence estimates because intermittent shedding can make stool-based testing insensitive. Strongyloidiasis is most common in tropical and subtropical environments with poor sanitation. In the United States, it is commonly reported among refugees and immigrants; in the 1980s, studies in the rural southeastern United States also reported prevalence estimates ranging from 1.2%–6.1% (1,2). Prevalence might have since decreased because of investments in sanitation (3); however, no recent studies have been done, and strongyloidiasis is not a reportable disease in any state.

The Kentucky Department for Public Health and CDC sought to determine whether *Strongyloides* transmission continues in a rural area of the United States where transmission has been demonstrated in previous serostudies. Kentucky is a state where strongyloidiasis historically has been endemic (2). In 2011, Kentucky had 15 strongyloidiasis-related hospital discharge diagnoses reported by the Healthcare Cost and Utilization Project database (4). Origin and travel history are not reported in that database, making country of exposure unclear for those cases. Approval for this project was obtained from the Kentucky Cabinet for Health and Family Services Institutional Review Board prior to the start of the study. Investigators recruited a convenience sample of patients attending a nongovernmental organization’s weekend clinic offering dental, vision, and medical services in southeastern Kentucky. All patients were eligible to enroll in the study and were referred for free treatment if needed. Patients provided informed consent, demographic information, exposure history, and blood samples that were tested by CDC for anti-*S. stercoralis* antibody by enzyme immunoassay; a positive result indicated current infection (titers decrease after successful treatment).

A total of 752 patients attended the clinic. Testing was offered in a public area frequented by all patients, and multiple invitations for testing were issued in group waiting areas. A total of 102 (13.6%) patients, all adults, agreed to be tested. Five patients tested positive for *S. stercoralis* antibody, including one man and four women, ranging in age from 21 to 69 years. All were born in the United States and provided addresses in one of four cities in southeastern Kentucky. Four had an indoor flush toilet; the fifth had an indoor toilet with manual waste removal. No travel to tropical countries was reported.

Although antibody testing cannot be used to differentiate between acute and chronic infection, given the lack of travel history, autochthonous transmission of *Strongyloides* appears to persist in this Appalachian area. Wider investigations are planned.
